# Regulatory mechanisms of cytoneme-based morphogen transport

**DOI:** 10.1007/s00018-022-04148-x

**Published:** 2022-02-04

**Authors:** Christina A. Daly, Eric T. Hall, Stacey K. Ogden

**Affiliations:** 1 Department of Cell and Molecular Biology, St. Jude Children’s Research Hospital, 262 Danny Thomas Pl. MS340, Memphis, TN 38105 USA; 2 St. Jude Graduate School of Biomedical Sciences, St. Jude Children’s Research Hospital, 262 Danny Thomas Pl, MS 1500, Memphis, TN 38105 USA

**Keywords:** HH, WNT, BMP, FGF, Signaling filopodia, Signal transduction

## Abstract

During development and tissue homeostasis, cells must communicate with their neighbors to ensure coordinated responses to instructional cues. Cues such as morphogens and growth factors signal at both short and long ranges in temporal- and tissue-specific manners to guide cell fate determination, provide positional information, and to activate growth and survival responses. The precise mechanisms by which such signals traverse the extracellular environment to ensure reliable delivery to their intended cellular targets are not yet clear. One model for how this occurs suggests that specialized filopodia called cytonemes extend between signal-producing and -receiving cells to function as membrane-bound highways along which information flows. A growing body of evidence supports a crucial role for cytonemes in cell-to-cell communication. Despite this, the molecular mechanisms by which cytonemes are initiated, how they grow, and how they deliver specific signals are only starting to be revealed. Herein, we discuss recent advances toward improved understanding of cytoneme biology. We discuss similarities and differences between cytonemes and other types of cellular extensions, summarize what is known about how they originate, and discuss molecular mechanisms by which their activity may be controlled in development and tissue homeostasis. We conclude by highlighting important open questions regarding cytoneme biology, and comment on how a clear understanding of their function may provide opportunities for treating or preventing disease.

## Overview

Organ and tissue development rely on coordinated dispersal of morphogen signals from cellular organizing centers that provide instructional cues to govern cell fate. Signaling proteins contributing to tissue morphogenesis include Hedgehog (Hh/HH) family members, Transforming Growth Factor-β (TGF- β) and Bone Morphogenic Protein (BMP) family members, WNTs, NOTCH, and members of the Epidermal Growth Factor (EGF) and Fibroblast Growth Factor (FGF) families. These molecules direct distinct transcriptional programs in target cells, oftentimes through concentration- and signal duration-dependent manners [1–5].

Establishment of terminal cell fate across different tissues results from coordinated input from different combinations of morphogen signals. For example, limb development and digit specification are orchestrated through WNT, FGF, BMP, and HH signaling, craniofacial development is instructed primarily by HH and WNT activity [6–8], and pancreatic and endocrine system development are dictated by FGF, BMP, HH, NOTCH, and WNT signals [9]. One of the best examples of the multi-faceted roles of signaling molecules is found in central nervous system (CNS) development. Here, HH, WNT, FGF, and BMP collectively signal to dictate cell fate at early developmental stages and to induce proliferation, promote cell survival, and guide axon pathfinding at later developmental stages [10–22].

A tractable genetic model system in which coordinated developmental signaling can be studied is provided by the *Drosophila* wing imaginal disc, a sac-like epithelial tissue that is composed of several types of progenitor cells that communicate with one another to drive wing morphogenesis. Epithelial cells of the wing disc signal to a segment of the tracheal system called the Air Sac Primordium (ASP), which develops into the adult dorsal air sacs. Myoblasts found in this tissue form the adult flight muscles, which are provided oxygen by the air sacs. For each of these components of the flight system to develop properly, numerous pathways must be induced in precise temporal- and tissue-specific manners. Pathways active during wing cell specification include Hh, the BMP homolog Decapentaplegic (Dpp), FGF homolog Branchless (Bnl), WNT homolog Wingless (Wg), and the NOTCH ligand Delta [23–27]. Dysregulation of any of these pathways during wing morphogenesis results in overt patterning defects that compromise wing development, underscoring the importance of organized multi-pathway input for proper fate determination.

In addition to their crucial contributions during development, morphogens also play key roles promoting tissue homeostasis. Morphogens act in the stem cell niche to maintain stemness and promote proliferation or differentiation in response to distinct cues. This is exemplified by maintenance of the intestinal epithelium. Repopulation of cells in the crypts occurs through continuous regeneration from stem cells located at the crypt base. WNT and NOTCH signals maintain the undifferentiated state of cells in the stem cell niche and BMPs promote differentiation of intestinal epithelial cells to replace dying cells in the crypts [28]. Indian Hedgehog (IHH) is expressed by differentiating epithelial cells in the midcrypt region where it signals to the mesenchymal intestinal stem cells (ISCs) [29]. IHH pathway activation indirectly reduces WNT-mediated proliferation, thus stimulating ISC differentiation [29]. Similar mechanisms are found in homeostasis and repair of other adult tissues including the brain, skin, prostate, and bladder [30]. Given the myriad of important functions that depend upon proper activity of these signaling proteins, it is not surprising that their dysregulation can lead to developmental disorders and cancers [31–36].

Despite the importance of controlled signal deployment and delivery for tissue development and homeostasis, the precise mechanisms by which morphogens and growth factors are transported between sending and receiving cells remain a topic of debate. Interest in the mechanisms by which morphogens travel from a signaling source was spurred by discovery of the Spemann–Mangold organizer. This pioneering work demonstrated that specific regions of a developing embryo could influence development of other embryonic regions upon their transplantation [37]. Subsequently, the Dalcq–Pasteels hypothesis suggested that signals emanating from organizing tissues would form a double gradient, with one set of molecules spreading from the vegetal pole of the embryo and a second set simultaneously forming from the dorsal cortex [38]. In 1952, the term “morphogen” was coined to describe the molecules making up these developmental maps [39]. The French Flag representation of morphogenetic gradient formation was proposed to explain how cell fate is established by dynamic equilibrium of exposure to signals across a developing tissue. In this model, a tri-colored flag design depicts how positional information is conferred upon fields of cells across a concentration gradient [40]. Source cells produce morphogens which organize into these gradients to dictate positional information [1]. These gradients can stretch to be quite broad, which allows for tissue patterning to be instructed at significant distances from a morphogen source. For example, in the developing limb bud, signals from the zone of polarizing activity (ZPA) can reach cells situated as far as 200 μm away from the signaling source [41]. How such long-range signals reach their intended targets is not yet clear, but has been proposed to occur through processes including free and assisted diffusion, cell-to-cell transfer through repeated cycles of endocytosis and exocytosis (transcytosis), or direct engagement of membrane-associated signaling molecules [1, 42–44].

While diffusion of signals from organizing tissues is the simplest model for gradient formation, many mature signals are insoluble due to the presence of transmembrane domains or specific lipid modifications that promote their association with signal-producing-cell membranes. For example, NOTCH ligands are transmembrane proteins that remain tethered to their site of production, and thus require direct cell–cell interaction for receptor–ligand engagement and signal activation [45]. HH family members are modified by both cholesterol and long-chain fatty acid modifications [46–48] and WNT family members harbor a palmitoylation modification [49, 50]. A recent study on FGF transport in the *Drosophila* wing imaginal disc revealed that the Bnl/FGF is GPI-anchored to the producing-cell membrane, requiring direct contact with receiving cells to activate signaling through its receptor Breathless (Btl) [51]. These observations, along with the inability of passive diffusion models to fully account for the stringency and robustness observed across physiological morphogen gradients, suggest a requirement for a mode of direct, contact-mediated morphogen delivery to target cells [52–54].

Two models have been proposed to address this need. The first is planar transcytosis, in which morphogens are directly transferred between neighboring cells through repeated cycles of receptor-mediated endocytosis, membrane recycling, and exocytosis [42, 55]. In this model, degradation dynamics of endocytosed signaling molecules in the signal-receiving cells directly dictate the concentration gradient across target tissues. Although this process allows membrane-tethered and insoluble morphogens to forgo travel through the extracellular space, it does not fully account for longer range signaling that is seen during physiological development in tissues such as the limb [52].

A second model, which is gaining increasing experimental support, postulates that morphogens travel between signal-producing and -receiving cells along specialized filopodia called cytonemes [43]. These actin-based, cell surface protrusions emanate from morphogen-producing and/or -receiving cells and act as molecular highways for direct exchange of developmental signaling molecules. Since their characterization in 1999, there has been an explosion in interest that has led to numerous advances in our understanding of cytoneme biology. Interrogation of cytoneme-mediated signaling in the developing *Drosophila* wing disc identified pathways using cytonemes to include Hh, Dpp/BMP, Bnl/FGF, Spitz/EGF, Wg/WNT, and NOTCH [43, 56–61]. *Drosophila* tissues in which cytonemes have been observed include the wing disc, abdominal epidermis, female germline stem cell niche, and the developing eye [43, 59–61].

Evidence supporting involvement of cytonemes in morphogen transport in vertebrate systems is also mounting. Sonic Hedgehog (SHH)-containing cytonemes have been documented in the developing chick limb bud and WNT-transporting cytonemes have been identified in zebrafish embryos [62–65]. Excitingly, a recent study demonstrated the presence of SHH-transporting cytonemes with lengths up to 80 μm in the Axolotl blastema during limb regeneration, suggesting involvement of the structures in cellular response to injury [66]. In vitro analysis of cytoneme function has also increased, providing opportunities for interrogating the molecular mechanisms that control cytoneme behavior. Recent mechanistic investigations have focused on SHH-mediated cytoneme formation using cultured murine cells [67] and WNT-transporting cytonemes have been studied in murine intestinal organoids [63, 65]. The expansion of models available for cytoneme research provides opportunities to identify protein partners contributing to cytoneme formation and signal transport along them.

## Cytonemes as specialized signaling filopodia

Decades ago, long, thin cellular protrusions were observed in developing sea urchin larvae and hypothesized to be involved in cellular motility [68, 69]. Years later, examination of the structures during sea urchin gastrulation led to the hypothesis that they did not influence cell motility, but instead facilitated cell–cell communication [70]. This hypothesis was strengthened by studies in *Drosophila* wing imaginal discs, which revealed thread-like projections extending from signaling centers toward fields of responding cells. Like the long filopodia observed in sea urchin, these structures, called cytonemes, were determined to be actin-based and highly dynamic. Furthermore, cytonemes could be induced ex vivo by exposing explants of *Drosophila* wing imaginal discs and mouse limb buds to an FGF source, supporting that they were responsive to extracellular cues. Because *Drosophila* cytonemes were observed to extend from organizing centers expressing Hh and Dpp/BMP, they were proposed to facilitate direct delivery of morphogens from signal-producing to signal-responding cells [43].

A persistent question about the cytoneme model centers around how they differ from the well-studied conventional filopodia that aid in cellular communication, motility, and wound healing. Conventional filopodia are actin-based membrane extensions that are generally ~ 0.1–0.3 μm in diameter and rarely reach lengths greater than 10 μm [71]. A variety of conventional filopodia with specialized functions have been described. These include myopodia (neuromuscular synapse formation), podosomes (matrix degradation), and invadopodia (cancer cell invasion) [72–74]. Despite differences in their functions, sizes, and behaviors, these protrusions all share a core structure of bundled actin filaments and extend from the cell surface following regulation by actin- and cytoskeletal-associated proteins [72–74]. Although cytonemes have similar diameters to these conventional filopodia, they have the potential to extend to significantly longer lengths. For example, anterior/posterior (A/P)-oriented cytonemes in *Drosophila* wing discs demonstrate average lengths of greater than 20 μm [75]. Incredibly, cytonemes extending from wing discs grown in culture have been documented to extend up to 700 μm to contact co-cultured, signal-responding cells [43]. This ability to grow to significant lengths, coupled with their morphogen cargo and distinct polarization relative to cellular organizing centers, has resulted in cytonemes being classified as a specialized type of cellular extension [43, 76]. Whether cytonemes are appropriately classified as specialized filopodia, or if they represent a new class of cellular extensions that are specifically suited to grow to incredible lengths to shape morphogen gradients, are open and controversial questions in developmental biology. Moreover, other cellular extensions including tunneling nanotubes (TNTs), intercellular bridges (IBs), and airinemes share both physical and functional characteristics with cytonemes.

TNTs are actin-based membrane protrusions with a diameter of 0.05–0.7 μm that can span lengths of several cell diameters [77, 78]. TNTs are reported to form through two different mechanisms, the first being cytoneme-like where a filopodium directed toward a neighboring cell makes a stable contact. The second mode of TNT formation occurs when two cells in direct membrane contact migrate away from each other while maintaining a point of contact through a TNT bridge [77, 79, 80]. Plasma membrane and cytoplasmic components are transferred freely between the connected cells through the bridge, thereby establishing a direct cytoplasmic connection. TNTs can also expand to facilitate vesicular transfer of individual vesicles and multi-vesicular bodies (MVBs). TNTs also allow for exchange of organelles including mitochondria, which provide a potential mechanism for energy replenishment during generation of these long structures [77, 78, 81]. Once formed, TNT bridges facilitate Ca^2+^ signaling and electrical synchronization between cells to promote downstream signaling in the contexts of development and immune cell communication. Studies of chick and quail cranial explants have shown that during development, migrating neural crest cells form intercellular TNTs for electrical coupling [82–84]. In the immune system, macrophages and T cells use TNTs for Ca^2+^ signaling, natural killer cells lyse their targets via perforin-containing TNTs, and dendritic cells use them to promote inflammation [80, 85–87]. In addition, TNTs formed by immune cells can be hijacked by Human Immunodeficiency Virus (HIV) and prion proteins for more efficient transmission [79, 88, 89].

The second type of cellular connection that shares similarity with the cytoneme is the IB. IBs, which are also referred to as ring canals, are similar in appearance to cytonemes and TNTs due to their ability to reach lengths of up to 350 μm [90]. They were first documented in spermatids and continue to be observed primarily in germ cells [91–95]. IBs are important for spermatogenesis and fertility and, like TNTs, are implicated in molecular and organelle transport and Ca^2+^ signaling [95–97]. The process of IB formation is what sets them apart from other actin-based structures. They are remnants of incomplete cytokinesis or syncytia, and as such, can be much thicker than filopodia with diameters ranging between 0.2 and 10 μm [98].

Airinemes are cytoneme-like signaling extensions reported to be crucial for NOTCH signaling during patterning of zebrafish stripes. Stripes are generated by two types of pigment-producing cells: yellow pigment-producing xanthophores and black pigment-producing melanophores. Melanophores express the receptor NOTCH, while xanthophores produce the NOTCH ligand DELTA [99]. Both are transmembrane proteins that require direct interaction between communicating cells. NOTCH receptor–ligand engagement promotes lateral inhibition, such that signal-sending cells instruct their signaling partners to adopt a different fate [45]. In this case, NOTCH signaling both communicates a survival signal and promotes clearance of melanophores from the inter-stripe region, resulting in accurate spatial patterning of the stripes [99, 100]. For airinemes to communicate over a distance, a xanthophore first forms a large DELTA-containing membrane bleb that is positive for the phospholipid phosphatidylserine (pS) [100, 101]. A nearby macrophage recognizes the pS-containing membrane and engulfs the bleb, forming an airineme vesicle [101]. The macrophage then migrates up to 189 μm away to deposit the large, signal-containing vesicle on the surface of an NOTCH-expressing melanophore [101].

Notch-mediated lateral inhibition occurring between distantly localized signaling partners is also observed in *Drosophila* where Delta-expressing cells in the wing disc activate Notch receptors on nonadjacent cells through transient cytoneme-like filopodia to instruct spatial patterning of sensory bristles [57, 58]. Filopodia-mediated, long distance Notch signaling also occurs in ovarian germ cells in flies, which use the structures to deliver Delta to distantly localized somatic cells to induce stem cell niche formation [102]. Whether the *Drosophila* Notch filopodia are true cytonemes or fly airineme-like structures has not yet been firmly established.

Despite sharing some physical and functional characteristics with TNTs, IBs, and airinemes, key differences set cytonemes apart from these other signaling extensions (Table 1). First, cytonemes do not appear to facilitate direct cytoplasmic transfer as is observed for TNTs and IBs. As will be discussed below, they are thought to provide signaling information by forming synapse-like connections with signal-receiving cells. A second difference is that, due to their narrow diameter, cytonemes are generally too thin to facilitate transfer of large organelles, so do not appear to transfer mitochondria like TNTs [103]. Airinemes differ from cytonemes, because they require both microfilaments and microtubules, while cytonemes do not [100]. Nevertheless, all these structures play important roles in cell-to-cell communication, underscoring the need for direct, contact-based signaling in development and tissue homeostasis.

**Table 1:** Cellular extensions contributing to cell–cell communication

	Cytonemes	Tunneling nanotubes	Intercellular bridges	Airinemes
Diameter	~ 200 nm	50–700 nm	200 nm- 10 μm	< 1 μm
Length	4–700 μm Average 10–25 μm	10–200 μm	Up to 350 μm	~ 10–250 μm
Cell/tissue type	Developing and adult tissues Stem cell niche	Migrating neural crest cells Immune cells: macrophages, T cells, natural killer (NK) cells, dendritic cells	Germ cells Developing tissues	Extend from Xanthophores to melanophores
Formation	Extension toward cell signaling partners	Extension toward neighboring cell Contact maintained between cells migrating apart	Incomplete cytokinesis/ syncytia	Macrophage engulfs membrane bleb, migrates to deposit on signaling cell
Function	Morphogen and growth factor transport Maintenance of stem cell niche Tissue homeostasis	Exchange of organelles Ca^2+^ signaling Electrical synchronization NK-mediated cell lysis, inflammation	Spermatogenesis and fertility Molecular/organelle transport Ca^2+^ signaling	Zebrafish stripe patterning
References	43, 56, 59–67, 75, 76, 104, 105, 106	77–87	90–98	99–101, 107

## Mechanisms of cytoneme initiation

Despite improved imaging technologies and the growing number of model organisms in which cytonemes have been observed, the precise molecular mechanisms by which cytonemes are initiated remain unclear. Intriguingly, both in vitro and in vivo studies indicate that morphogen expression can promote cytoneme initiation. Transient overexpression of SHH, the NOTCH ligand JAGGED, FGF2, or WNT3A in mouse NIH3T3 cells increases cytoneme occurrence rates [67, 108]. Similar observations have been reported in *Drosophila* where Dpp overexpression leads to increased numbers of cytonemes extending from the A/P organizer of the larval wing disc [75] . Dpp also influences cytoneme directionality. Whereas cytonemes of control discs projected specifically toward A/P and dorsal/ventral (D/V) axes, cytonemes induced by ectopic Dpp grew outwards in all directions [75]. The conserved ability of morphogen signaling proteins to induce, stabilize, and/or guide cytonemes suggests an ability of morphogens to communicate with cytoskeletal regulators in signal-sending cells. The specific signals facilitating this communication are not yet known. However, the molecular mechanisms driving traditional filopodia initiation may provide clues about how cytonemes initiate in morphogen-expressing cells.

### Contributions of GTPases to filopodial extensions

**Fig. 1:**
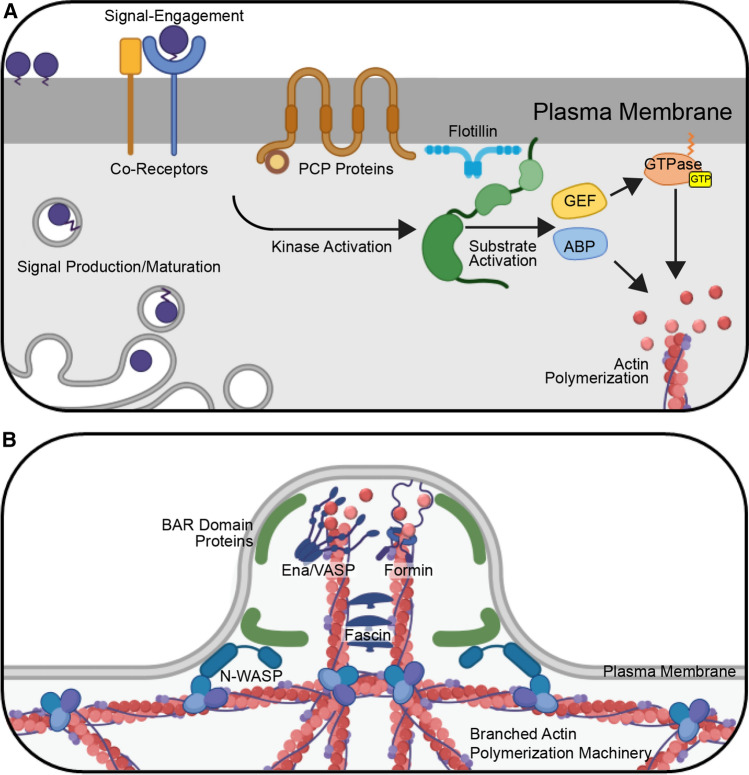
Initiation of Cytoneme Formation. **A** Morphogen initiation of cytoneme budding. Following maturation and secretory transport, morphogens engage cognate deployment proteins and co-receptors at the cell surface to initiate intracellular signaling through effector kinases and GTPases. Kinases activate substrates including Actin-Binding Proteins (ABPs) and Guanine nucleotide Exchange Factors (GEFs), which in turn promote GTP binding to small GTPases. GTPase effectors polymerize actin at these sites. For WNT signaling, Planar Cell Polarity (PCP) proteins contribute to deployment. Flotillin is frequently detected at sites of morphogen clustering, suggesting that it may contribute to aggregation of transmembrane and lipid-modified molecules involved in this process. **B** Cytoneme extension. Following activation of actin-polymerization machinery, actin is assembled into linear bundles that expand the cell surface to form a cytoneme bud. BAR domain-containing proteins are activated by Cdc42 to induce membrane curvature and concentrate actin machinery at these sites. In these cases, BAR–domain protein interactor Wiskott-Aldrich Syndrome Protein (N-WASP) promotes branched actin assembly to provide a scaffold for linear actin filament assembly. The unbranched actin polymerization, accomplished by Ena/VASP family members and/or formin proteins, then acts to extend the nascent cytoneme. Linear actin filaments are cross-linked upon binding by Fascin

Filopodia can be initiated by activation of cell surface receptors that recruit and activate cytoskeleton polymerization machinery. Recruitment is typically orchestrated by rapid and specific activities of kinases and GTPases downstream of the receptors [109] (Fig. 1a). Rapid responses are achieved, because effector kinases, GTPases, and downstream formins that control actin polymerization are poised for signal-induced activation [109, 110]. The Rho GTPase family member Cdc42 is the most common GTPase effector regulating filopodia initiation [110, 111]. GTPases are commonly activated by guanine nucleotide exchange factors (GEFs) that promote the exchange of GDP for GTP on the small G protein [112] (Fig. 1a). GEFs can be activated by cytokines, growth factors, GPCR ligands, proteoglycans, and integrins to stimulate Cdc42 at the site of receptor engagement [112].

Once activated, GTPases associate with the plasma membrane where they interact with downstream effectors including kinases, scaffolding proteins, and actin nucleation and polymerization machinery to orchestrate cytoskeletal reorganization for filopodial extension [110, 113] (Fig. 1a). A prominent Cdc42 GTPase effector in the nucleation of actin in filopodia is the membrane-binding, F-BAR domain protein Transducer of Cdc42-dependent Actin assembly (TOCA-1), which binds to Cdc42-GTP at sites where outgrowth will occur [114] (Fig. 1b). The subsequent formation of filopodia occurs in a stepwise process, starting with recruitment and activation of molecules that exist in autoinhibited conformations within the cytoplasm [114]. First, TOCA-1 recruits and activates Wiskott-Aldrich syndrome protein (N-WASP), an actin nucleation promoting factor [115]. Upon activation, N-WASP recruits the actin-nucleating ARP2/3 complex, which accepts and incorporates actin monomers into growing polymers [116]. This results in nucleation of a branched actin structure at the site of filopodial outgrowth [114] (Fig. 1b). Formins and/or Enabled/vasodilator-stimulated phosphoprotein (Ena/VASP) family members can attach to the growing ends of actin filaments at this site and polymerize linear filaments to form plasma membrane protrusions that establish nascent filopodial structures [109, 114, 117] (Fig. 1b). In the final step, the actin cross-linker protein Fascin bundles the parallel actin filaments during extension of the growing filopodium [114] (Fig. 1b).

### Pathway-specific cytoneme initiation

It is reasonable to hypothesize that morphogens with the ability to impact cytoneme occurrence might tap into actin-regulatory pipelines to promote cytoneme initiation and growth. The specific cytoskeletal regulators engaged are likely dictated by the morphogen being transported, which may act through receptors, co-receptors, and/or adaptor proteins at the cell surface. In the case of HH, studies in both murine and *Drosophila* models indicate that HH, its deployment protein Dispatched (Disp/DISP), and co-receptors Interference Hedgehog/Cell adhesion-associated, Down-regulated by Oncogenes (Ihog/CDON) and Brother of Ihog/Brother of CDON (Boi/BOC) can all promote cytoneme occurrence [67, 108, 118, 119]. Disp overexpression in cultured *Drosophila* cells promotes cytonemes by slowing retraction rates of Hh-containing extensions [108]. In cultured murine cells, expression of BOC or CDON increases cytoneme occurrence rates to a level comparable to what is observed upon SHH overexpression. DISP forms a complex with SHH and BOC or CDON in cytonemes that is essential for ligand delivery through the structures, such that loss of either DISP or BOC/CDON decreases cytoneme-based SHH signal delivery to target cells [67]. As such, HH may signal through DISP/co-receptor complexes in ligand-producing cells to boost cytoneme occurrence and/or stability for efficient delivery to cellular targets. Consistent with this hypothesis, both BOC and CDON have been reported to interact with ABL tyrosine kinase, which is linked with increased filopodia persistence in fibroblasts [120–122]. During neurite outgrowth, the ABL–BOC interaction results in SHH-mediated c-Jun N-terminal Kinase (JNK) activation [121]. During myogenesis, the ABL–CDON interaction activates p38MAPK via scaffold protein JNK-interacting protein 4 (JIP4/JLP) [122].

JNKs are established regulators of actin that are activated during various developmental programs to remodel the cytoskeleton for stress fiber formation, microtubule stabilization, axon regeneration, regulation of smooth muscle contractility, and cellular migration [123]. Treatment of murine embryonic dorsal root ganglia neurons with an actin depolymerizing agent promotes JNK activation to restore cytoskeletal integrity and facilitate axon regeneration [124]. Moreover, in rat cortical neurons, JNK activation results in formation of mature filopodia by phosphorylating the actin cross-linker MARCKS-like protein 1 (MARCKSL1), which in turn facilitates actin bundling [125].

Despite these capabilities, the role of JNK activity in regulation of cytonemes remains unclear. In *Drosophila*, JNK activation appears to be inhibitory toward formation of Hh-containing cytoneme-like projections in the hematopoietic niche [126]. In this context, loss of the transcription factor Relish, which represses JNK, leads to filopodial loss and ‘trapping’ of Hh in the niche. This occurs due to increased activity of JNK effectors Ena and Fascin/Singed, which play key regulatory roles in actin polymerization and bundling [126]. However, Fascin activity is required for formation of cytonemes by mammalian embryonic stem cells [127] and the family of Ena/VASP proteins effectively promote filopodia formation in multiple cellular contexts [128]. As such, cytoneme formation likely relies on a balance of signaling activity by these effectors.

In addition to being able to impact actin dynamics through kinase activation, BOC and CDON have also been reported to influence cytoskeletal dynamics by promoting GTPase activation. During commissural axon guidance, the GEF regulator Engulfment and Cell Motility (ELMO) binds DOCK3/4 GEFs to stabilize their interaction with Rac1-GTPase for Rac1 activation. In the absence of SHH, BOC sequesters ELMO and prevents ELMO-mediated Rac1 activity. However, upon axon exposure to SHH, BOC binding to ELMO is blocked to allow for Rac1-GTP binding for cytoskeletal remodeling to induce growth cone turning toward the SHH source [129]. Studies in C2C12 myoblasts suggest that CDON may also be able to influence actin remodeling by activating the GTPase Cdc42 [130]. This is thought to occur through CDON binding the Cdc42 regulator BNIP-2, which binds both Cdc42 and ARHGAP1 to promote Cdc42-GTP binding during myogenesis [131]. However, in vivo studies suggest that SHH does not signal through Cdc42 to influence cytonemes in the chick limb bud, because conditional inactivation of the GTPase did not disrupt cytoneme formation in the limb bud mesenchyme [62]. A lack of Cdc42 involvement is also reported for FGF-induced cytonemes in cultured mesenchymal cells. Instead, the atypical GTPase RhoD is activated downstream of FGF2/4/8 to induce its effector formin mDia3C to promote N-WASP-independent actin polymerization [132]. Formins are also involved in cytonemes in the *Drosophila* wing disc where Diaphanous (Dia) promotes cytoneme-mediated Dpp signaling in the ASP [104].

Recent reports suggest that WNT uses similar strategies to influence cytoneme dynamics for its transport. WNT binding to the receptor tyrosine kinase ROR2 is important for cytoneme initiation in zebrafish embryos, murine intestinal organoids, and human cancer cell lines [63]. Like what is observed for DISP, BOC, and CDON, ROR2 overexpression increases cytoneme incidence in fibroblasts. In these cells, WNT8A clusters co-localized with ROR2 at the plasma membrane prior to cytoneme initiation from the cluster sites [63]. WNT–ROR2 clusters are hypothesized to work by concentrating actin regulators for filopodial extension at sites of ligand enrichment and may also improve efficiency of WNT8A presentation at receiving-cell membranes to ensure robust signal initiation [63, 105]. Mechanistic studies suggest ROR2 promotes cytoneme extension by recruiting planar cell polarity (PCP) proteins and Casein Kinase 1 (CK1) to the plasma membrane. This facilitates CK1-mediated phosphorylation of the PCP protein VANGL2 (Van-Gogh-like). WNT8A, ROR2, and VANGL2 are then loaded into cytoneme buds where they stimulate JNK activation to promote cytoneme outgrowth in a manner that is dependent upon the I-BAR domain protein IRSp53 [63, 65]. In rat hippocampal neuronal cultures, JNK3 is recruited to the actin cytoskeleton upon WNT7A treatment to promote axon branching and increased motility of axonal filopodia [133]. Because these JNK effects on WNT-mediated cytoneme formation differ from what is seen with Hh cytonemes in the *Drosophila* hematopoietic niche [126], it is likely that the impact of JNKs on cytoneme formation varies based on morphogen and tissue contexts.

In addition to working through JNK, WNT has also been proposed to initiate cytoneme formation through the Rho GTPase Cdc42 in a PCP-dependent manner [134]. WNT-ROR2-induced cytonemes of zebrafish fibroblasts require Cdc42 activity, because introduction of dominant-negative Cdc42 led to a significant loss in cytoneme occurrence and WNT8A signal initiation in receiving cells [63]. In this context, Cdc42 activation led to actin nucleation and polymerization by N-WASP and IRSp53 [134]. A potential avenue for activation of these intracellular signaling molecules is suggested by the discovery that WNT co-receptors, LGR4 and LGR5, can induce cytoneme formation in Human Embryonic Kidney (HEK) cells [135]. The long protrusions induced by LGR5 are positive for Fascin and are enriched for VASP at their tips [135]. As such, the ability of LGR4/5 to influence cytoneme outgrowth may indicate that cytoneme formation occurs downstream of ligand engagement with these co-receptors.

Another molecule that has been linked to morphogen-specific cytoneme initiation in the *Drosophila* wing disc is Flotillin-2/Reggie-1 (Flo2), which marks cholesterol-rich plasma membrane microdomains [59, 106]. Flo2 is present in Hh-containing wing disc cytonemes where it contributes to proper cytoneme length and Hh gradient establishment [59]. The size of the gradient of Wg in the *Drosophila* wing disc is also reported to depend upon Flo2, because its knockdown resulted in a shortened range of Wg dispersion and compaction of target gene expression [136]. Flotillins function as protein scaffolds for clustering tyrosine kinases, Rho GTPases, and adhesion molecules to regulate cellular polarity, extracellular matrix interaction, and cytoskeletal regulation [137]. Consistent with Flo2 involvement in cytoneme formation in *Drosophila*, overexpression of its homolog, FLOT2, in mammalian epithelial cell lines can induce filopodia-like cell surface protrusions [138]. It is tempting to speculate that Flotillins promote the clustering of molecules involved in cytoneme formation at specific membrane microdomains that are enriched for molecules with post-translational modifications including GPI-linkage, cholesterolylation, palmitoylation, and myristoylation (Fig. 1a). These modifications are common on GTPases, kinases, and some morphogens, and can potently influence their interaction with the plasma membrane [137]. Recruitment of cytoskeletal regulation machinery where signals are accumulating on the cell surface could then promote focused cytoneme outgrowth at these sites.

## Cytoneme extension and stabilization

One of the most striking characteristics of dynamic cytonemes is their length variability. In the *Drosophila* wing disc, ASP cytonemes range from 12 to 50 μm, with an average length of ~ 23 μm [61]. Cytonemes extending from myoblasts are approximately 25 μm long and cytonemes from apical epithelia are on average ~ 20 μm, but can reach lengths of over 80 μm [56, 75]. Similar dynamic ranges are found in vertebrates. Cytonemes in zebrafish embryos average between 10 and ~ 17 μm and cytonemes extending from cells of the developing chick limb bud can range from 34 to 150 μm [62, 63, 134]. To date, murine cytonemes have only been analyzed in cultured cells, and range in length from 4 μm to upwards of 50 μm [65, 67, 132]. As such, cytoneme lengths are likely tissue- and context-dependent.

The precise molecular mechanisms governing cytoneme extension in differing cell and tissue contexts are not known. However, mutagenesis studies suggest that formins are likely responsible for the long, flexible nature of cytonemes due to their function in nucleating unbranched actin filaments and preventing capping of filament ends [109]. Perturbations of actin-regulatory proteins including Capping Protein (CP), SCAR/WAVE, or Pico/Lamellipodin shortens cytonemes formed by Ihog-expressing wing disc cells from an average length of over 60 μm to under 30 μm [59]. Moreover, the activated form of the *Drosophila* formin Dia enriches in tips of cytonemes extending from the ASP and is required for effective communication between the ASP and wing disc epithelium [104]. Loss of actin-regulatory proteins can lead to collapse of the Hh morphogen gradient across the developing wing disc, supporting that regulation of cytoneme length through actin modulation directly impacts morphogen spread [59].

In addition to being influenced by cell autonomous actin modulation, cytoneme length is also impacted by interactions with components of the extracellular matrix (ECM). In *Drosophila*, loss of PCP proteins Van Gogh (Vang) and Prickle (Pk) significantly decreases lengths of FGF-containing cytonemes that extend between the ASP and underlying wing disc [139]. This was attributed to alteration of ECM composition, which showed reduced levels of the matrix protein laminin and the ECM heparan sulfate proteoglycans (HSPGs) Division abnormally delayed (Dally) and Dally-like protein (Dlp). HSPG disruption prevented cytoneme extension and pathfinding, suggesting crucial roles for the ECM in promoting cytoneme action [139]. Moreover, cytonemes from cells of the *Drosophila* wing disc fail to extend over tissue that is deficient for glypican biosynthesis, further supporting potent modulation of cytoneme extension by PCP and ECM proteins [59]. Communication between the ECM and PCP proteins appears to be an evolutionarily conserved mode of controlling cytoneme length. Recent work in vertebrate model systems supports a role for VANGL2 in driving actin polymerization in WNT-containing cytonemes, because its overexpression in cultured zebrafish fibroblasts led to an approximate 187% increase in cytoneme lengths [65]. ECM components are similarly important during zebrafish development, as evidenced by the HSPG Glypican-4 (GPC4) being required to maintain cytoneme lengths in the WNT5B- and WNT11F2-producing endoderm [140].

Two hypotheses have been proposed to explain how the ECM promotes cytoneme extension. The first suggests that the ECM stores attractants for cytoneme pathfinding akin to how the ECM influences axon guidance [139]. This is best described by the model of restricted diffusion, in which secreted molecules interact with ECM proteins like HSPGs to establish an extracellular gradient for navigation through the tissue [141]. A second hypothesis is that GPI-anchored glypicans of the HSPG family facilitate direct interactions between the ECM and cytoneme membranes and serve as substrates upon which cytonemes can travel [139]. The HSPGs Dally and Dlp cluster on the cell surface of cultured *Drosophila* cells and Dlp clusters on Hh-transporting cytonemes [142]. In the *Drosophila* wing disc, Dally and Dlp interact with and stabilize ectopically expressed Ihog on cytoneme membranes by slowing cytoneme elongation and retraction velocities [143]. Similarly, BOC and CDON enrich in microdomains along portions of chick limb bud cytonemes that remain static, suggesting that they bind to extracellular components to stabilize the structures as they extend [62].

## Morphogen loading and transport

**Fig. 2:**
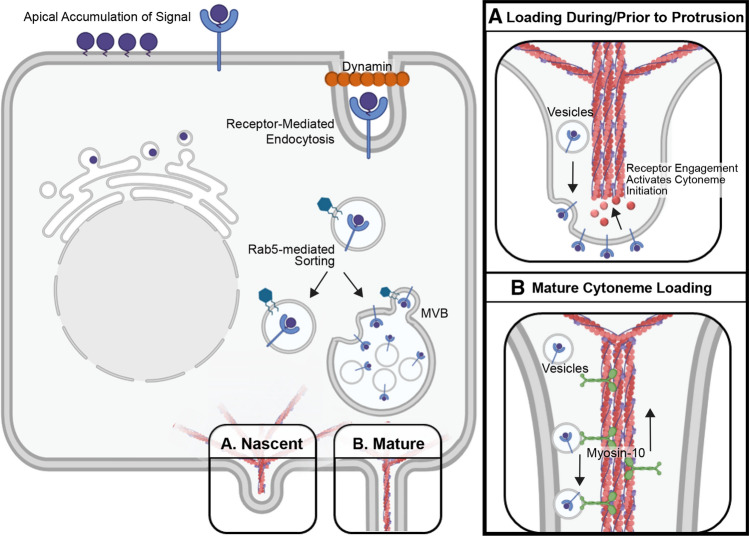
Morphogen Loading and Transport. **A** Morphogen enrichment in budding cytonemes. In polarized cells, morphogens accumulating on apical membrane are recycled in a dynamin-dependent manner for sorting to basal cytonemes. The vesicles are sorted by Rab GTPases directly to basolateral membrane for cytoneme loading through accumulating at sites of budding cytonemes or for incorporation into multi-vesicular bodies for active transport along cytoneme extensions. **B** Morphogen entry into existing cytonemes. Vesicles containing signaling proteins are loaded into mature cytonemes for transport to cytoneme tips by Myosin-10 (MYO10)

Models explaining how morphogens load into cytonemes include free diffusion from the plasma membrane onto cytoneme membrane, extension from the plasma membrane at sites of ligand accumulation, endocytic recycling for vesicular and multi-vesicular body (MVB)-mediated entry, and active transport (Fig. 2) [63, 67, 106, 144].

### Contributions of morphogen diffusion and membrane clustering to cytoneme loading

In the *Drosophila* ASP*,* the FGF family member Bnl has been observed to scatter along the surface of cytonemes where it is tethered by a GPI anchor, suggesting that it enters through diffusion along the membrane [51, 144]. Similarly, SHH-N, which receives an amino-terminal lipid modification but lacks a carboxyl-terminal lipid attachment, can localize to the extracellular face of cytoneme membranes in chick limb buds, suggesting that it may also enter cytonemes passively through membrane association [62]. However, studies of dually lipid-modified HH proteins in the *Drosophila* wing disc and cultured mouse cells revealed localization of the morphogen inside vesicular structures, raising the possibility that specific lipid modifications may impact cytoneme loading behavior [67, 106].

Studies carried out in zebrafish embryos demonstrate that WNT8A-ROR2 form clusters at the plasma membrane that initiate cytoneme formation, allowing for their incorporation into the nascent cytoneme tip during outgrowth [63]. These WNT8A-positive accumulations co-localize with TOCA-1 at the plasma membrane prior to protrusion of a cytoneme bud [134]. WNT ligand remains at the tips of cytonemes during outgrowth, supporting a model in which actin-regulatory signaling downstream of ligand accumulation promotes localized actin assembly [63, 134]. This allows for protrusion of the ligand-containing portions of the plasma membrane in the form of cytoneme outgrowth, resulting in efficient, direct delivery of the morphogen to distant cells (Fig. 2a).

### Contribution of endocytosis to cytoneme loading

Mechanistic interrogation of morphogen membrane recycling suggests that the endocytic pathway may play a role in cytoneme loading of some signaling proteins. Studies in *Drosophila* polarized epithelia revealed that Dpp-, Hh-, and Wg-containing vesicles all have features indicative of having undergone receptor-mediated endocytosis from the apical cell surface [145, 118, 146] (Fig. 2). Dpp and its receptor Thickveins (Tkv) co-localize with the endocytic marker human transferrin receptor (hTfR) in basolateral puncta in wing epithelia [145], and Wg signals are reported to transfer through the wing disc epithelium via internalized vesicles called argosomes [146, 147]. In the signal-producing cells of the *Drosophila* wing disc, Hh localizes to basolateral MVBs that provide exovesicles that carry ligand to signal-receiving target cells [106] (Fig. 2). MVB formation and vesicular trafficking rely on activity of the endosomal sorting complex required for transport (ESCRT) protein complexes I, II, and III [148]. ESCRT protein Vps32 labels Hh-containing exovesicles found in the extracellular space of *Drosophila* wing discs, and depletion of various ESCRT family members attenuates long-range Hh signaling [149]. Similarly, activity of ESCRT-III protein CHMP1A promotes SHH release in extracellular vesicles during mouse brain development to ensure proper neural patterning [150]. Disp, Ihog, and Dlp are reported to enrich with Hh in exovesicles within the *Drosophila* wing disc, and DISP and CDON/BOC enrich with SHH in vesicles inside cultured mouse cell cytonemes [67, 106]**.** Additional support for involvement of endocytic recycling in priming cells to send a long-range signal is provided by the *Drosophila* ASP and wing disc where Dynamin, which pinches off the endocytic bud, is required for endocytic recycling of both Hh and Dpp. Accordingly, its mutation alters Hh gradient formation in vivo [118, 104, 151] (Fig. 2).

Consistent with the important role of membrane recycling for Hh deployment, the transporter-like protein Disp, which neutralizes the membrane-tethering activity of Hh lipid modifications, stimulates recycling endocytosis of Hh proteins from the apical surface of ligand-producing cells in the *Drosophila* wing disc [152]. Disp-Hh complexes that internalize from apical membrane then target back to the membrane in an Rab4/5-dependent manner to release Hh for long-range signaling [118, 152] (Fig. 2). Importantly, cytonemes that contribute to long-range signaling across polarized epithelial tissue typically originate from basolateral membranes, and Hh signal-receiving cells of the developing wing disc endocytose Hh in complex with its receptor Ptc from basolateral membrane [56, 58, 118, 119, 153]. As such, Disp likely contributes to long-range signaling through redirecting apical Hh to basolateral membranes for cytoneme loading. Consistent with this hypothesis, Disp loss ablates long-range Hh signaling activity, but does not disrupt short-range juxtacrine signaling in the wing imaginal disc [118, 154]. Intriguingly, the apical-basal distribution of cellular extensions is controlled by a gradient of active Rac1-GTPase, with higher activity occurring in basolateral compartments and correlating with filopodia and lamellipodia formation [155]. It remains to be seen if Rac1 activity is required for basolateral cytoneme extension, and whether Disp may signal to Rac1 to promote Hh cytoneme activity. Notably, in mice, Rac1 is activated to induce cytoskeletal remodeling by noncanonical SHH signaling in ligand-receiving cells [156], suggesting that Hh may be able to link with this small G protein in signal-producing cells to influence cytoneme growth.

Recycling of Hh prior to cytoneme entry suggests a model whereby lipid-modified morphogens first accumulate on the plasma membrane where they interact with packaging and release machinery that promotes their basolateral retargeting for long-range signaling [118] (Fig. 2). Consistent with this hypothesis, in the *Drosophila* wing disc lipid-modified Wg proteins that signal at short range occur apically, while long-range signals are sent basolaterally [157]. However, unlike Hh, Wg does not rely on Dynamin for its recycling, which may indicate that individualized modes of membrane recycling and ligand transport exist for different morphogen signals [151]. Interestingly, a recent study suggests that intracellular trafficking of specific morphogens can provide control over the amount of morphogen permitted to exit a signal-producing cell. In this study, graded increases in expression of Dpp, Hh, or Wg in the *Drosophila* wing disc failed to affect wing disc morphology, because consistent levels of ligand were targeted to basolateral membrane regardless of production level [158]. As such, intracellular trafficking machinery may ensure that appropriate concentrations of morphogen are packaged and redistributed for cytoneme-mediated transport.

### Contributions of active transport to cytoneme signaling

The actin-based motor Myosin 10 (MYO10) enriches at cytoneme tips with SHH-N in the developing chick limb bud and with cholesterol-modified SHH in cultured mouse cells, suggesting that it promotes SHH movement to cytoneme tips [62, 67] (Fig. 2b). Both in vitro and in vivo studies support a requirement for MYO10 in SHH signal propagation, because cultured mouse cells lacking MYO10 or expressing a cargo-binding-deficient mutant fail to effectively enrich SHH in cytonemes or induce a response in signal-receiving cells. Consistent with these observations, neural tubes of MYO10-deficient mice show reduced *Gli1* induction and patterning defects indicative of attenuated SHH morphogen gradient activity [67]. However, while there is support for MYO10 involvement in SHH transport in vertebrate systems, *Drosophila* lack an MYO10 homolog, suggesting that a different molecular mechanism may drive Hh transport along fly cytonemes [159]. Nevertheless, evidence for a conserved role for MYO10 in cytoneme-based morphogen transport in vertebrates is mounting. In the developing zebrafish neural plate, MYO10 colocalizes with WNT8A and cargo-receptor EVI-WLS at cytoneme tips where they accumulate to promote pathway activation in receiving cells [134]. MYO10 has also been observed to traffic along LGR5-induced cytonemes in HEK cells, suggesting a conserved mechanism of active transport [135].

## Morphogen reception by target cell cytonemes

**Fig. 3:**
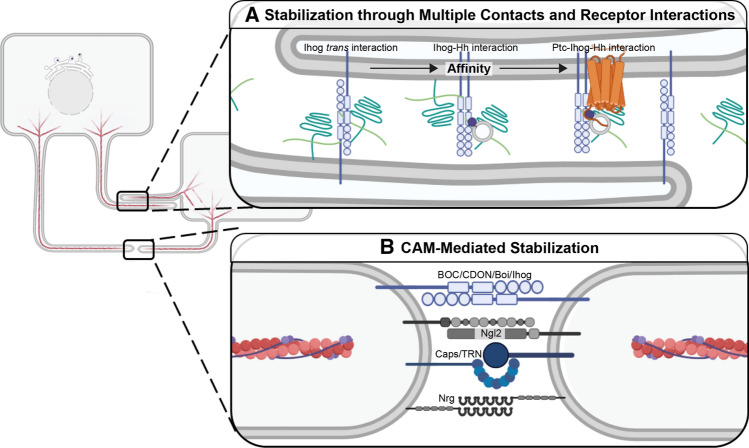
Stabilization of Cytoneme Contacts. **A** Cytoneme stabilization through additive contact. Stability can be conferred by protein–protein interactions *in trans*. Cytoneme-localized transmembrane proteins can bind heparan sulfate proteoglycans (HSPGs) (green). For Hh (dark purple), HSPGs contribute to Ihog (blue) interactions *in trans* to stabilize cytoneme-cytoneme contacts (left). HSPGs in the ECM or on cytoneme membrane can also cooperate in the higher affinity Ihog–Hh interaction (center). Highest affinity interactions are achieved between the Ihog, ligand, and Hh receptor Ptc (orange) to allow for morphogen transfer and pathway activation. **B** Stability of cytoneme contact through CAMs. HH co-receptors BOC/Boi and CDON/Ihog also function as CAMs to stabilize these points of interaction. Dpp transport is supported by wing disc expression of CAM Capricious (Caps)/Tartan (Trn) and by ASP expression of neuronal CAMs Neuroligin 2 (Ngl2) and Neuroglian (Nrg) at cytoneme tips

**Fig. 4:**
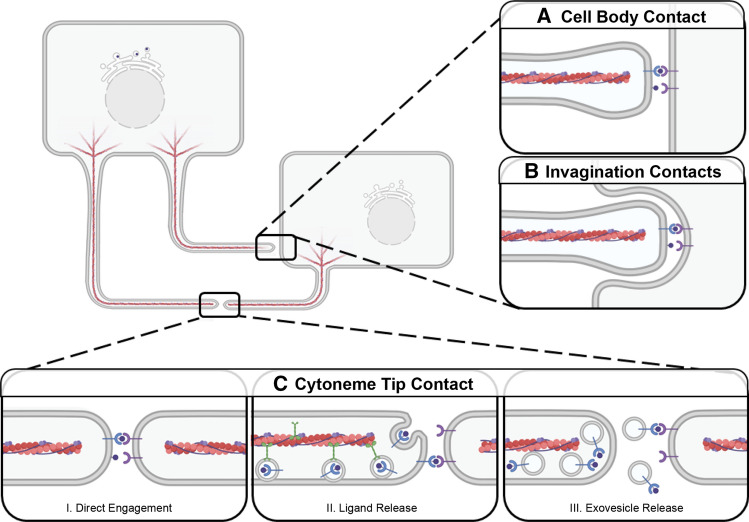
Morphogen Reception by Target Cells. Cytonemes can deliver signal through contact with receiving-cell bodies (**A**,**B**) or other cytonemes (**C**). **A** Signal-containing cytonemes directly contact the cell body. **B** Cytonemes extend into plasma membrane invaginations on the cell body. **C** Cytoneme tips contact to form a morphogenetic synapse where signals are presented through: (I) Incorporation into the plasma membrane at the cytoneme tip. (II) Transport along cytonemes for release from the tip. (III) Vesicular transport along the cytoneme for exovesicle-based release at a morphogenetic synapse for signaling

Cytonemes extend from both signal-sending and signal-receiving cells and are thought to transfer morphogens through direct contact at morphogenetic synapses [54, 160, 161]. Cytoneme contact has been observed in both fixed and live cellular imaging in fly and vertebrate models [67, 104]. Live imaging studies reveal that cytoneme associations can occur as transient connections suggestive of scan and release activity, or stable connections that may be reinforced by adhesion protein or co-receptor functions [59, 62, 67, 162] (Fig. 3). Connections can occur between cytoneme tips or through cytoneme–cell body association, but the regulatory events determining how contact is made are not yet clear (Fig. 4). Whereas live imaging of murine NIH3T3 cells overexpressing SHH and MYO10 revealed stable contact between cytoneme tips [67], analysis of Ihog-stabilized cytonemes in the *Drosophila* wing disc using GFP reconstitution across synaptic partners (GRASP) revealed multiple cytoneme-cytoneme contact points along the lengths of the extensions [162] (Fig. 3a). In the latter study, homophilic Ihog interactions worked *in trans* to stabilize multiple discrete connections, indicating that enrichment of specific proteins in cytonemes can influence connection behavior [162].

Signal-sending cytonemes can also directly contact signal-receiving-cell bodies to extend into plasma membrane invaginations. This has been observed in cultured mouse cells and in vivo in *Drosophila* [67, 103] (Fig. 4b). It is not yet clear how a mode of cytoneme contact is selected. It is possible that cytoneme-to-cell body contact is favored when producing-cell cytonemes outnumber cytonemes extending from receiving cells. Cytoneme insertion into receiving-cell plasma membrane invaginations may also be favored in situations requiring delivery of higher concentration of morphogen to achieve a desired signaling threshold.

To receive a cytoneme-based signal, a target cell must express the proper receptor to confer responsiveness to morphogens enriched in the delivering cytoneme. In the *Drosophila* ASP*,* the receptors for the ligands Dpp, Spitz/EGF, and Bnl/FGF segregate to distinct signal-receiving cytonemes that only interact with the signal-sending cytonemes that contain their cognate ligand [61, 104]. However, ASP cytonemes that contain Btl are also positive for Ptc, suggesting that some signal/receptor clustering can occur [163].

During somite formation in chick, the WNT receptor Frizzled 7 (FZD) enriches in puncta on cytoneme-like structures originating from the WNT-responsive dermomyotome [164], and in flies, Fzd-positive myoblast cytonemes orient directly toward Wg-producing cells in the wing disc [56]. Furthermore, the Dpp receptor Tkv and the Hh receptor Ptc enrich in puncta that move along cytonemes [75, 119]. In some tissues, the density of cytonemes from signal-receiving cells appears to shift relative to signal strength and receptor enrichment. For example, in the *Drosophila* ASP, Btl/FGFR-containing cytonemes form a gradient across signal-receiving tissue, such that cytoneme numbers decrease as distance from the Bnl/FGF source increases [144]. As such, signal-receiving-cell cytonemes constitute an important part of concentration gradient establishment.

Cytonemes of both signal-sending and signal-receiving cells are proposed to identify targets through sensing chemoattractant signals. This was first demonstrated in *Drosophila* where cytonemes of the wing imaginal disc extended toward the signaling center located at the A/P border [43]. More recent studies of dynamic cytonemes suggest a “random walk” model in which cytonemes orient in a deliberate manner through stabilization of connections that form between compatible molecules [76]. A mathematically generated model of WNT transport supports that random walk behavior has the capacity to rapidly form a robust concentration gradient that recapitulates what occurs in developing tissues [165]. The dynamic nature of cytonemes increases the likelihood that morphogen-enriched filopodial tips will encounter receptor-enriched cytonemes or cell membrane through which they establish stable contact. Cytoneme dynamics are influenced by rapid bursts of extension and retraction from the originating cell body [165]. In the conventional filopodia, extension and retraction dynamics are controlled through a balance between the functions of actin assembly and disassembly machinery. Similar mechanisms are likely in place to control cytoneme dynamics, because in the chick limb bud, Cofilin enriches at tips of Fascin-containing cytonemes. Its retrograde movement toward the cell body correlates with rapid retraction of dynamic cytonemes in this tissue [62].

Once successful contact is made between a dynamic cytoneme and a target cell cytoneme or membrane, the interaction may be stabilized to allow for signaling to occur (Fig. 3). For the Hh pathway, the adhesion protein Ihog and the HSPGs Dally and Dlp are thought to be critical for stable contact between Hh-sending and -receiving cytonemes in the *Drosophila* wing disc [119] (Fig. 3a). Ihog was first demonstrated to function in Hh signal reception by acting as a co-receptor with Ptc in signal-receiving cells [166–169]. More recently, its function as a cell adhesion molecule (CAM) was demonstrated to promote longevity of cytoneme contacts to ensure Hh gradient establishment in the wing discs [59, 162, 169]. Ihog contributes to these activities through both hetero- and homo-typic interactions that are governed by binding affinity [162]. Lower affinity Ihog–Ihog interactions promote cytoneme-to-cytoneme association and high-affinity Ihog–Hh interactions promote morphogen transfer [162] (Fig. 3a). The highest affinity interactions occur through the heterotypic Ptc–Ihog–Hh complex, which drives associations that lead to pathway activation in signal-receiving cells [162] (Fig. 3a). This model suggests that cytoneme pathfinding may occur through establishment of ligand–co-receptor and ligand–receptor interactions that improve efficiency of signal transport.

Although this type of competitive binding coordination has not yet been reported for other signaling pathways, cytonemes containing other morphogens are reported to use CAMs to stabilize cytoneme attachments in a manner similar to what occurs with neuronal synapses. For example, Dpp-containing cytonemes from the *Drosophila* wing disc are stabilized by the presence of the CAM Capricious (Caps) or its paralog Tartan (Trn) at their tips (Fig. 3b). Disruption of Caps or Trn function leads to defective Dpp uptake and signaling in the ASP, leading to altered morphogenesis [104]. Additional proteins common to neuronal synapses including the adhesion protein Neuroligin 2 (Nlg2) and the neuronal CAM Neuroglian (Nrg) are also reported to play roles in Dpp cytoneme synapse function (Fig. 3b). Knockdown of either Nlg2 or Nrg in the signal-receiving ASP decreases cytoneme number, which results in alteration of ASP morphology [104, 170].

In murine embryonic stem cells (ESCs), WNT-receiving cytonemes are stabilized through engagement of co-receptors LRP5/6 [127]. Similar mechanisms are in place for FGF signal transmission, because a GPI-anchored form of the *Drosophila* FGF Bnl at ASP cytoneme tips binds its receptor Btl in a CAM-like manner to stabilize synapses. Removal of the domain of Bnl facilitating CAM activity blocked formation of long, polarized cytonemes from both source and recipient cells, suggesting Bnl–Btl binding facilitates forward and reverse signaling to reinforce cytoneme function [51].

**Fig. 5:**
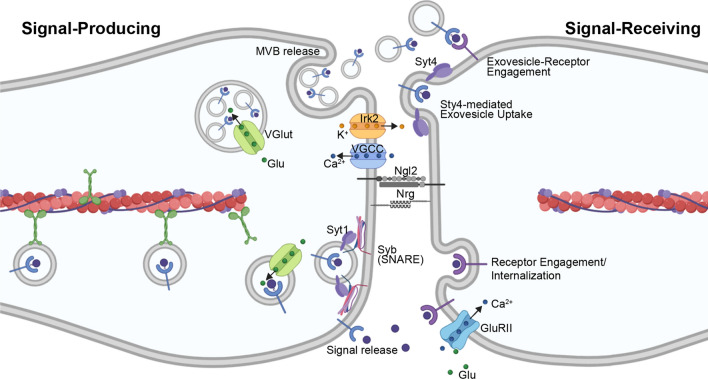
Glutamatergic Signaling at Morphogenetic Synapses. Cytonemes from a signal-producing cell are primed for signaling through uptake of glutamate molecules (green circles) through the Vesicular Glutamate Transporter (VGlut). At the cytoneme membrane, Ca^2+^ ions (blue circles) are imported by the Voltage-Gated Calcium Channel (VGCC) and K^+^ ions (orange circles) are exported by Inward-Rectifying K^+^ Channel (Irk2) to establish an ion gradient. The Ca^2+^-binding protein Synaptotagmin 1 (Syt1) targets signal-containing vesicles to the plasma membrane for vesicle docking by Ca^2+^-dependent R-SNARE family members. Release of vesicular contents (signals and/or exosomes) at the synapse results in release of glutamate into this site. Glutamate binds the non-NMDA ionotropic glutamate receptor (GluRII) to promote its activity for Ca^2+^ uptake by the signal-receiving cell. Ca^2+^-binding Synaptotagmin 4 (Syt4) functions on the extracellular surface of the signal-receiving cytoneme to facilitate signal reception. The neuronal synaptic adhesion protein Neuroligin 2 (Nlg2) and neuronal CAM Neuroglian (Nrg) function on the ASP to stabilize this interaction

Further similarity between cytonemes and neuronal signaling is highlighted by a recent report demonstrating that trafficking of Tkv-containing puncta along ASP cytonemes is regulated by Ca^2+^ [170]. The Dpp-producing wing disc relies on Ca^2+^ signaling generated by the Voltage-Gated Calcium Channel (VGCC) and inward-rectifying K^+^ channel (Irk2). These ion gradients promote activity of Synaptotagmin Ca^2+^-binding protein 1 (Syt1) to target Dpp-containing vesicles toward the plasma membrane, where Ca^2+^-dependent Synaptobrevin (Syb), an R-SNARE family member, mediates vesicle docking for Dpp release [170, 171]. The Dpp-containing, pre-synaptic vesicles from wing discs contain glutamate, which is taken up by the vesicular glutamate transporter (VGlut). Glutamate release promotes postsynaptic Ca^2+^ uptake by ASP cytonemes via the non-NMDA ionotropic glutamate receptor (GluRII). Uptake of Ca^2+^ by the signal-receiving cytonemes then stimulates activity of Synaptotagmin 4 (Syt4) to promote receptor internalization for pathway activation [170] (Fig. 5). Evidence of glutamatergic signaling in mammalian cytonemes has been recently established in murine ESCs [127]. This cell population extends cytonemes toward WNT-producing trophoblast stem cells (TSCs) for self-renewal [127]. Upon formation of a stable cytoneme contact, ionotropic glutamate receptor (iGluR) generates a Ca^2+^ flux within the ESC cytoneme [127]. As such, intracellular signaling activity once thought to be specific to neurons may be more broadly employed to allow for morphogen signaling in developing tissues and stem cell compartments [172].

## The future of cytoneme research

As summarized above, the cytoneme-based model for morphogen transport continues to gain experimental support. There is much research effort focused on identifying the mechanisms controlling cytoneme initiation, morphogen loading, pathfinding, and signal delivery, because several questions remain unanswered. Key among them are defining when and where cytonemes initiate and determining how cytoneme behavior is tuned between signal-sending and -receiving cells in organizing tissues to ensure pattern formation. An additional aspect of cytoneme biology that is likely to be an intense area of research focus going forward will be to define the series of events that occur following signal reception by a receiving-cell cytoneme. Is signal initiated immediately upon receptor binding at a cytoneme tip, or do ligand/receptor complexes return to the cell body to signal from discrete membrane locations or vesicles, as has been proposed for the WNT signalosome [173]? Notably, for HH and WNT pathways, the co-receptors BOC/Boi, CDON/Ihog, and ROR2, function in both producing and receiving cells, suggesting that shared protein partners between connecting cytonemes may enhance signal transfer [63, 65, 67]. Determination of how other morphogen classes ensure successful signal transfer will require further research.

As it becomes increasingly clear that cytonemes are crucial for dispersion of many types of morphogens and growth factors, an understanding of the underlying molecular mechanisms governing cytoneme behavior will be paramount. The aspects of cytoneme regulation that are universally conserved during initiation, elongation, and stabilization, and activities that are morphogen-specific must be determined.

Due to the central role morphogens play during embryogenesis, it is likely that cytoneme dysfunction contributes to developmental disorders. Thus, a clear understanding of the regulatory mechanisms controlling their activity may provide novel opportunities for prevention or treatment of devastating developmental syndromes. Cytoneme biology is also likely relevant to cancer progression. EGFR and RET tumor models in *Drosophila* reveal decreased tumor growth and increased survival when cytoneme formation is disrupted in neoplastic cells and surrounding tissue [174]. In humans, many tumor types express and secrete morphogens that are thought to contribute to tumor–stroma communication during tumor growth and metastasis [175]. Because morphogen expression in tumors can drive cytoneme formation [67, 75], it is possible their high-level expression may facilitate direct lines of communication between cancer cells and surrounding tissue to facilitate tumor growth. In astrocytoma, microtubule-containing extensions are observed between tumor cells and documented to function as routes for tumor invasion and proliferation [176]. These results lend credence to the idea that cytonemes not only act as highways for morphogen transport in development and tissue homeostasis, but can be coopted in pathological progression.

## Data Availability

Not applicable. This is a review of published literature.
